# 

**Published:** 1876-07

**Authors:** 


### Contents of July Number.

### ORIGINAL.

ART. I.—The Surgical Treatment of Dysmenorrhœa. By O. H. E. Clarke, M. D. .......... 441

ART. II.—Abstract of the Proceedings of the Buffalo Medical Association, 466

### EDITORIAL DEPARTMENT.

International Medical Congress. .......... 469

Close of Volume Fifteen .......... 470

American Dermatological Association .......... 471

BOOKS REVIEWED:

Filth Diseases and their Prevention. By John Simon, M. D.,.. 472

A Treatise on Diseases of the Nervous System. By Wm. H. Hammond, M. D., .......... 474

Books and Pamphlets Received, .......... 474

